# Effects of different energy and protein levels on growth performance, carcass traits, and meat quality of Pekin ducks

**DOI:** 10.3389/fvets.2025.1438526

**Published:** 2025-03-19

**Authors:** P. Y. Zhao, X. Ao, W. Zhao, L. Zhou

**Affiliations:** ^1^Faculty of Quality Management and Inspection & Quarantine, Yibin University, Yibin, China; ^2^Sichuan Higher Education Engineering Research Center for Agri-Food Standardization and Inspection, Yibin University, Yibin, China; ^3^Key Laboratory of Sichuan Province for the Utilization of Solid State Fermentation Resources, Yibin University, Yibin, China; ^4^Tie Qi Li Shi Group Co., Mianyang, China

**Keywords:** energy, protein, growth performance, taste, duck

## Abstract

This study was conducted to determine the effects of different energy and protein levels on growth performance, carcass traits, meat quality, and taste scores of Pekin ducks. A total of 1,800 numbers of 15-day-old ducks (10 replicate pens, 60 birds/pen, 819 ± 18 g/duck) were blocked based on body weight (BW) and randomly allotted to three treatments with different metabolizable energy (ME) and crude protein (CP) levels in this 28-day experiment. Dietary treatments were as follows: (1) low energy protein (LEP), 3,150 kcal/kg and 16% for the grower diet and 3,250 kcal/kg and 15% for the finisher diet; (2) medium energy protein (MEP), 3,250 kcal/kg and 16.5% for the grower diet and 3,350 kcal/kg and 15.5% for the finisher diet; (3) high energy protein (HEP), 3,350 kcal/kg and 17% for the grower diet and 3,450 kcal/kg and 16% for the finisher diet. During days 15–28, body weight gain (BWG) in the LEP group was higher (*p* < 0.05), whereas FI, F/G, and caloric conversion were lower (*p* < 0.05) in the MEP and HEP groups. During days 29–42, birds fed with LEP and HEP diets had lower (*p* < 0.05) BWG but higher F/G and caloric conversion compared with those fed with the MEP diet. Birds fed with the LEP diet had higher (*p* < 0.05) BWG than those fed with MEP and HEP diets throughout the experiment. The abdominal fat yield in the LEP group was lower (*p* < 0.05) than in the HEP group. Birds fed with the LEP diet had higher (*p* < 0.05) left breast meat yields than those fed with MEP and HEP diets. The roasting loss in the LEP and HEP groups was lower (*p* < 0.05) than in the MEP group. Birds fed with LEP and HEP diets had higher (*p* < 0.05) comprehensive scores, flavor scores, scent scores, and taste scores, but lower color scores compared with those fed with the MEP diet. In summary, the LEP diet with the lowest cost may be the most appropriate based on growth performance, roasting loss, and taste scores.

## 1 Introduction

The most common breed used to produce duck meat in the world is Pekin duck, a local species in China (1). Higher abdominal and subcutaneous fat levels are ideal for roasting Pekin ducks (2). Roast ducks require high-fat content during processing to ensure good flavor (3).

The determination of nutrient requirements in Pekin ducks is necessary to optimize production goals and to use the genetic potential. It is well documented that poultry carcass quality and adipogenesis can be affected by several factors, such as genetics, species, age, sex, dietary factors, and slaughtering and processing conditions (4, 5). Dietary nutrient density is the most critical factor, which may not only influence growth performance and carcass quality but also production costs (6, 7). Nevertheless, there is some discrepancy in nutrient density for best performance or flavor (8). A recent study showed that the dietary nutrient density required to achieve both the best growth performance and carcass traits in Pekin ducks for roasting might vary (9). The best body weight gain (BWG) and feed-to-gain ratio (F/G) were obtained when Pekin ducks were fed with high-apparent metabolizable energy (ME, 3,285 kcal/kg) and high crude protein (CP, 19%) diet from 15 to 35 days of age (8). Several studies have evaluated and established different optimal ME and CP levels in Pekin ducks for different production purposes based on growth performance and carcass quality during the starter and grower phases (1, 10–12). Furthermore, there was an interaction between ME and CP in the growth performance of Pekin ducks with different ME:CP ratios (8).

The carcass qualities, such as abdominal fat and subcutaneous fat amount and composition, are more important traits in Pekin ducks used for roasting. Dietary lipids such as fat and vegetable oils are usually added to Pekin duck diets to increase energy density and modulate lipid quality (13). It is generally reported that animal fats rich in saturated fatty acids are digested less easily than vegetable oils rich in unsaturated fatty acids (14, 15). Our previous study showed that Pekin ducks fed with duck fat had higher abdominal fat and subcutaneous fat yields than those fed with soybean oil and palm oil at the same nutritional level (2). However, little information was available on Pekin ducks fed with optimal ME and CP diets containing duck fat.

Undoubtedly, it is necessary to develop cost-effective duck diets containing optimal ME and CP levels using low-cost duck fat for Pekin ducks. Studies to evaluate the meat quality of Pekin roast ducks are limited. It was recommended that the requirement for ME and CP is 3,000 kcal/kg and 16% for Pekin ducks aged 15–42 years (16). We hypothesized that a low ME and CP diet is sufficient for the growth performance of Pekin roast ducks. Therefore, the objective of this study was to determine the influence of different ME and CP levels on growth performance, carcass traits, meat quality, and taste scores of Pekin ducks fed with diets containing duck fat with a constant ME:CP ratio and amino acid (AA) profile from 15 to 42 days of age. This can provide the appropriate ME and CP requirements for a Peking duck for roasting.

## 2 Materials and methods

### 2.1 Experimental design and duck husbandry

The Animal Welfare Committee of Yibin University (Yibin, Sichuan, China) approved the animal care protocol used in these experiments (Approval Number: YB2023045).

In this 28-day experiment, 1,800 Pekin ducks (Z-type crosses at 15 days of age) were randomly allocated to three treatments based on an average initial body weight (BW) of 819 ± 18 g. There were 10 replications (pens) per treatment and 60 ducks per pen (30 males and 30 females). All birds were housed in a commercial farm with stainless steel battery brooders in an environmentally controlled facility. The pens were equipped with feeders, nipple drinkers, and raised plastic floors. The environmental temperature and humidity were maintained at 22°C and 60%, respectively. A two-phase feeding program was used: a grower diet was offered from 15 to 28 days of age and a finisher diet from 29 to 42 days of age. All diets were formulated to meet all the nutrient requirements of ducks (NRC, 1994) (16), and the dietary treatments were as follows: (1) low energy protein (LEP), 3,150 kcal/kg and 16% for the grower diet and 3,250 kcal/kg and 15% for the finisher diet; (2) medium energy protein (MEP), 3,250 kcal/kg and 16.5% for the grower diet and 3,350 kcal/kg and 15.5% for the finisher diet; (3) high energy protein (HEP), 3,350 kcal/kg and 17% for the grower diet and 3,450 kcal/kg and 16% for the finisher diet (Tables 1, 2). The ME:CP ratio (197 for the grower diet and 216 for the finisher diet) and AA profile in each diet were kept constant. The birds were given free access to water and pellet feed throughout the experiment (Table 3).

**Table 1:** Diet composition (as-fed basis).

Items	Grower (days 15–28)			
	ME, kcal/kg	3,150	3,250	3,350
	CP, %	16.0	16.5	17.0
Ingredients, %				
Corn		46.69	43.03	39.69
Soybean meal (CP 45%)		1.46	3.38	5.18
Wheat flour (CP 14%)		10.00	10.00	10.00
Duck fat		4.00	6.00	8.00
Rice bran		4.62	4.40	4.11
Corn gluten meal (CP 60%)		—	—	—
Wheat germ		6.00	6.00	6.00
Peanut meal		5.00	5.00	5.00
Corn distiller-dried grains with soluble		14.00	14.00	14.00
Extruded full-fat soybean		3.00	3.00	3.00
Calcium phosphate		1.38	1.36	1.37
Limestone		1.36	1.35	1.34
Sodium chloride		0.32	0.32	0.32
L-Lysine HCl (70%)		1.31	1.24	1.17
DL-Methionine (99%)		0.19	0.19	0.19
Threonine (99%)		0.37	0.35	0.33
Vitamin premix^1^		0.15	0.15	0.15
Trace mineral premix^2^		0.15	0.15	0.15
Analyzed nutrient composition				
ME, kcal/kg^3^		3,150	3,250	3,350
Dry matter, %		88.6	88.7	88.9
CP, %		16.01	16.51	17.02
Ether extract, %		8.50	10.28	12.20
Crude fiber, %		3.26	3.22	3.21
Total ash, %		5.78	5.81	5.86
Lysine, %		1.25	1.25	1.25
Methionine, %		0.44	0.44	0.44
Threonine, %		0.85	0.85	0.85
Tryptophan, %		0.16	0.16	0.16
Calcium, %		0.90	0.90	0.90
Total phosphorous, %		0.64	0.64	0.64
ME:CP ratio^3^		197	197	197

1 Provided per kilogram of diet: vitamin A, 2,500 IU; vitamin D_3_,400 IU; vitamin E, 10 IU; vitamin K_3_, 0.5 mg; vitamin B_1_, 2.0 mg; vitamin B_6_, 2.5 mg; vitamin B_12_, 0.02 mg; nicotinic acid, 55 mg; pantothenic acid, 10 mg; folic acid, 1.0 mg; and biotin, 0.1 mg.

2 Provided per kilogram of diet: 60 mg Fe (FeSO_4_·7H_2_O); 8 mg Cu (CuSO_4_·5H_2_O); 60 mg Zn (ZnSO_4_·7H_2_O); 50 mg Mn (MnSO_4_·H_2_O); 0.1 mg Se (Na_2_SeO_3_·5H_2_O); and 0.2 mg I (KI).

3 Calculated values from the NRC (1994) data. ME, metabolizable energy; CP, crude protein.

**Table 2:** Diet composition (as-fed basis).

Items	Finisher (days 29–42)			
	ME, kcal/kg	3,250	3,350	3,450
	CP, %	15.0	15.5	16.0
Ingredients, %				
Corn		47.03	52.82	48.86
Soybean meal (CP 45%)		5.00	5.00	5.00
Wheat flour (CP 14%)		10.00	10.00	10.00
Duck fat		5.60	6.10	8.00
Rice bran		5.74	—	—
Corn gluten meal (CP 60%)		—	3.15	3.85
Wheat germ		6.00	3.00	4.40
Peanut meal		0.74	—	—
Corn distiller-dried grains with soluble		12.00	12.00	12.00
Extruded full-fat soybean		3.00	3.00	3.00
Calcium phosphate		1.42	1.53	1.50
Limestone		1.29	1.23	1.25
Sodium chloride		0.32	0.32	0.32
L-Lysine HCl (70%)		1.00	1.04	1.02
DL-Methionine (99%)		0.19	0.17	0.17
Threonine (99%)		0.37	0.34	0.33
Vitamin premix^1^		0.15	0.15	0.15
Trace mineral premix^2^		0.15	0.15	0.15
Analyzed nutrient composition				
ME, kcal/kg^3^		3,250	3,350	3,450
Dry matter, %		88.7	88.9	88.9
CP, %		15.02	15.50	16.01
Ether extract, %		10.00	9.72	11.50
Crude fiber, %		3.09	2.65	2.61
Total ash, %		5.75	5.30	5.34
Lysine, %		1.10	1.11	1.11
Methionine, %		0.44	0.44	0.44
Threonine, %		0.85	0.85	0.85
Tryptophan, %		0.15	0.15	0.15
Calcium, %		0.88	0.88	0.88
Total phosphorous, %		0.65	0.58	0.58
ME: CP ratio^3^		216	216	216

1 Provided per kilogram of diet: vitamin A, 2,500 IU; vitamin D_3_,400 IU; vitamin E, 10 IU; vitamin K_3_, 0.5 mg; vitamin B_1_, 2.0 mg; vitamin B_6_, 2.5 mg; vitamin B_12_, 0.02 mg; nicotinic acid, 55 mg; pantothenic acid, 10 mg; folic acid, 1.0 mg; and biotin, 0.1 mg.

2 Provided per kilogram of diet: 60 mg Fe (FeSO_4_·7H_2_O); 8 mg Cu (CuSO_4_·5H_2_O); 60 mg Zn (ZnSO_4_·7H_2_O); 50 mg Mn (MnSO_4_·H_2_O); 0.1 mg Se (Na_2_SeO_3_·5H_2_O); and 0.2 mg I (KI).

3 Calculated values from the NRC (1994) data. ME, metabolizable energy; CP, crude protein.

**Table 3:** Fatty acid composition and peroxide content of supplemental duck fat.

Items, % fatty acid content	Duck fat content, %
C12:0	0.12
C14:0	1.11
C16:0	21.2
C16:1	5.03
C18:0	7.11
C18:1	41.5
C18:2	20.8
C18:3	1.60
Total fatty acid content, %/EE	88.5
Peroxide value (mEq/kg)	1.62

### 2.2 Sample processing and laboratory analyses

Feed samples were analyzed for dry matter (Method 934.01), CP (Method 990.03), ether extract (Method 954.02), crude fiber (Method 978.10), total ash (Method 942.05), calcium, and phosphorus (Method 985.01) according to the standard procedures of the AOAC International (17). The AA content of all diets was determined using an amino acid analyzer (Biochrom 20, Pharmacia Biotech, Cambridge, England). Briefly, representative samples of the diets were hydrolyzed in 6 mol/L of hydrochloric acid for 22 h at 110°C under reflux conditions. For the determination of methionine and cystine, separate feed samples were oxidized with formic acid before hydrolysis and were then measured as methionine sulfone and cysteic acid. Tryptophan was determined by alkaline hydrolysis for 20 h at 110°C. All analyses were conducted in duplicate (18).

### 2.3 Sampling and measurements

The BW of each duck was weighed at 15, 28, and 42 days of age. The feed intake (FI) per pen was calculated daily. The BWG, FI, and F/G were then calculated as follows (19). Mortality was recorded as it occurred, and the weights of the dead birds were used to adjust the F/G ratio. The European production efficiency factor (EPEF) was calculated per pen as follows: $\text{EPEF}=\frac{\text{Survival rate}\;\left(\%\right)\times BW\;\left(\text{kg}\right)}{\text{Marketing}\;\text{age}\;\left(\text{days}\right)\times F/G}\times 100\%$.

At the end of the experiment, all ducks from each replicate were killed for evaluating carcass traits in a commercial factory (2). Feed was withdrawn 4 h before processing. Ducks were weighed and placed in transportation coops. These ducks were weighed and euthanized after electrical stunning (KY-TZLSX; Keyuan Mechanical Factory, Zhucheng, China), followed by exsanguination, defeathering, evisceration, and weighing again to obtain blood and feather weight. After the carcasses were stored on ice overnight, the cold carcass (without neck and feet), breast meat, skin, and subcutaneous fat were removed by trained personnel and weighed after flushing with saline. Carcass yield was determined as carcass weight in relation to BW and expressed as a percentage of BW (%), whereas blood, feather, breast meat, skin, subcutaneous fat, and abdominal fat yield were expressed as percentages of the carcass weight. The lightness (L^∗^), redness (a^∗^), and yellowness (b^∗^) values of the left skinless breast meat surface on the dorsal side were determined in triplicate under 100 Lux using a chroma meter (Minolta CR-410 Chroma Meter; Konica Minolta Sensing Inc., Osaka, Japan). The rip loss of breast meat was measured using approximately 2 g of muscle sample according to the plastic bag method (20). Both the left breast meat and subcutaneous fat were used to determine slice shear force (20). Briefly, the samples were cut parallel to the fiber orientation into pieces measuring 1 × 1 × 4 cm from the thickest portion of the cooked meat (60 min at 70°C in a water bath), and the shear force value was determined using a Rheometer (Fudoh-Rheo Meter RT-2005J; Rheotech Ltd., Tokyo, Japan) fitted with a 5 kg compression load cell with a crosshead speed of 30 cm/min. The peak force values, measured by the shearing of the centers of the cores perpendicular to the fibers, were used to determine the instrumental shear force values of the samples. The overall value was obtained from 12 measurements for each sample.

The roasting loss was determined as the difference between the carcass weight before and after roasting and was expressed as a percentage of carcass weight (2). Ten roasting ducks were selected from each pen for blind tasting. Blind tasting was used to evaluate the taste scores of breast meat after roasting by 10 trained panelists. The panelists were selected from the roasting duck restaurants and trained to evaluate the case scores until the rating difference was less than 10%. The comprehensive scores consist of color (30%), scent (10%), flavor (30%), and taste (30%) with different weighting coefficients. All the scores ranged from 1 to 10, and the higher score means the better sensory scores.

### 2.4 Statistical analysis

Data were analyzed by ANOVA (Analysis of Variance) using the GLM procedure of SAS (SAS Inst. Inc., Cary, NC, United States) (21) with the pen as the experimental unit. Differences between treatments were determined using Duncan’s multiple range test. The taste score data were analyzed using a multinomial model in PROC GENMOD. Variability in the data is expressed as the standard error of the means (SEM).

## 3 Results

### 3.1 Growth performance

During days 15–28, birds fed with the LEP diet had significantly (*p* < 0.05) higher BWG but significantly (*p* < 0.05) lower FI, F/G, and caloric conversion than those fed with MEP and HEP diets (Table 4). During days 29–42, BWG in the MEP group was significantly (*p* < 0.05) higher than in the LEP and HEP groups. Birds fed with the HEP diet had significantly (*p* < 0.05) lower FI than those fed with LEP and MEP diets. Compared with the LEP and HEP groups, F/G and caloric conversion were significantly (*p* < 0.05) lower in the MEP group. Overall, BWG was increased in the LEP group compared with the MEP and HEP groups. No differences were observed in FI, F/G, and EPEF among treatments during days 15–42.

**Table 4:** Effects of different metabolizable energy (ME) and crude protein (CP) levels on the growth performance of Pekin ducks^1^.

Item^2^	ME, kcal/kg	LEP 3,150 3,250	MEP 3,250 3,350	HEP 3,350 3,450	SEM^3^	*p-*value
	CP, %	16.0 15.0	16.5 15.5	17.0 16.0		
Initial BW (kg)		0.82	0.82	0.82	0.01	0.89
Final BW (kg)		3.69^a^	3.68^ab^	3.65^b^	0.01	0.04
Days 15–28						
BWG, kg		1.37^a^	1.21^c^	1.33^b^	0.01	0.02
FI, kg		2.93^c^	2.98^b^	3.01^a^	0.01	0.01
F/G		2.14^c^	2.47^a^	2.26^b^	0.02	0.03
Caloric conversion^4^		6.73^c^	8.03^a^	7.80^b^	0.04	0.03
Days 29–42						
BWG, kg		1.50^b^	1.66^a^	1.51^b^	0.01	0.04
FI, kg		3.91^a^	3.87^a^	3.77^b^	0.02	0.03
F/G		2.61^a^	2.34^c^	2.49^b^	0.02	0.03
Caloric conversion^4^		8.47^a^	7.83^b^	8.41^a^	0.04	0.02
Days 15–42						
BWG, kg		2.87^a^	2.86^ab^	2.83^b^	0.01	0.04
FI, kg		6.84	6.85	6.77	0.02	0.13
F/G		2.38	2.39	2.39	0.02	0.35
EPEF^5^		554	551	568	7.85	0.48

1 Means represent 10 replicates with 60 birds per cage (*n* = 10/group).

2 BWG, body weight gain; FI, feed intake; F/G, feed-to-gain ratio; EPEF, European production efficiency factor; LEP, 3,150 kcal/kg and 16% for the grower diet and 3,250 kcal/kg and 15% for the finisher diet; MEP, 3,250 kcal/kg and 16.5% for the grower diet and 3,350 kcal/kg and 15.5% for the finisher diet; HEP, 3,350 kcal/kg and 17% for the grower diet and 3,450 kcal/kg and 16% for the finisher diet.

3 Standard error of the means.

4 The caloric conversion was calculated using the following formula: Caloric conversion (Mcal/kg weight gain) = Dietary ME density (Mcal/kg) × Feed intake (kg) ÷ Weight gain (kg).

5 $\text{EPEF}=\frac{\text{Survival rate}\;\left(\%\right)\times BW\;\left(\text{kg}\right)}{\text{Marketing}\;\text{age}\;\left(\text{days}\right)\times F/G}\times 100\%$

### 3.2 Carcass traits

Birds fed with the HEP diet had significantly (*p* < 0.05) higher abdominal fat yields than those fed with the LEP diet (Table 5). The left breast meat yield in the LEP group was significantly (*p* < 0.05) higher than in the MEP and HEP groups. Birds fed with the MEP diet had significantly (*p* < 0.05) lower skin yield than those fed with the HEP diet. There were no differences in eviscerated carcasses, subcutaneous fat, left leg meat, blood, or feather yield among the treatments.

**Table 5:** Effects of different metabolizable energy (ME) and crude protein (CP) levels on carcass traits in Pekin ducks^1^.

Item, %^2^	ME, kcal/kg	LEP^3^ 3,150 3,250	MEP^3^ 3,250 3,350	HEP^3^ 3,350 3,450	SEM^4^	*p-*value
	CP, %	16.0 15.0	16.5 15.5	17.0 16.0		
Eviscerated carcass		72.36	72.99	73.27	0.68	0.22
Skin		35.91^ab^	35.36^b^	36.28^a^	0.23	0.04
Subcutaneous fat		37.72	37.66	38.39	0.30	0.33
Abdominal fat		2.47^b^	2.66^ab^	2.76^a^	0.04	0.03
Left breast meat		8.87^a^	8.04^b^	7.95^b^	0.08	0.02
Left leg meat		11.44	11.36	11.42	0.12	0.29
Blood		4.08	4.68	4.10	0.22	0.35
Feather		4.10	4.05	4.14	0.30	0.49

1 Means represent 10 replicates with 60 birds per cage (*n* = 10/group).

2 Carcass trait yield, %.

3 LEP, 3,150 kcal/kg and 16% for the grower diet and 3,250 kcal/kg and 15% for the finisher diet; MEP, 3,250 kcal/kg and 16.5% for the grower diet and 3,350 kcal/kg and 15.5% for the finisher diet; HEP, 3,350 kcal/kg and 17% for the grower diet and 3,450 kcal/kg and 16% for the finisher diet.

4 Standard error of the means.

### 3.3 Meat quality and taste scores

Dietary treatment did not affect lightness (L^*^), redness (a^*^), yellowness (b^*^), shear force, drip loss of breast meat, or shear force of subcutaneous fat (Table 6). The roasting loss in the LEP and HEP groups was significantly (*p* < 0.05) lower than in the MEP group. Birds fed with the MEP diet had significantly (*p* < 0.05) lower comprehensive and flavor scores than those fed with LEP and HEP diets. The overall appearance color score by panelists in the HEP group was significantly (*p* < 0.05) higher than that in the LEP and MEP groups. Birds fed with the MEP diet had significantly (*p* < 0.05) lower scent and taste scores than those fed with LEP and HEP diets.

**Table 6:** Effects of different metabolizable energy (ME) and crude protein (CP) levels on meat quality and taste scores in Pekin ducks^1^.

Item	ME (kcal/kg)	LEP^2^ 3,150 3,250	MEP^2^ 3,250 3,350	HEP^2^ 3,350 3,450	SEM^3^	*p-*value
	CP (%)	16.0 15.0	16.5 15.5	17.0 16.0		
Lightness (L^*^)		35.82	37.27	36.46	2.98	0.17
Redness (a^*^)		16.47	17.35	18.11	1.69	0.25
Yellowness (b^*^)		8.70	7.87	8.72	1.33	0.65
Shear force of breast meat (kg)		223.8	230.7	225.6	26.34	0.34
Shear force of subcutaneous fat (kg)		150.5	152.3	153.1	20.15	0.56
Drip loss, %		1.93	2.14	2.03	0.09	0.28
Roasting loss %		4.62^b^	6.04^a^	4.87^b^	0.05	0.02
Comprehensive score		7.55^a^	6.96^b^	7.33^a^	0.07	0.04
Color (30%)		8.00^b^	8.00^b^	8.17^a^	0.04	0.03
Scent (10%)		7.50^a^	6.50^c^	6.83^b^	0.06	0.02
Flavor (30%)		7.33^a^	6.67^b^	7.33^a^	0.05	0.01
Taste (30%)		7.33^a^	6.34^c^	6.67^b^	0.06	0.03

1 Means represent 10 replicates with 10 persons per group (*n* = 10/group).

2 LEP, 3,150 kcal/kg and 16% for the grower diet and 3,250 kcal/kg and 15% for the finisher diet; MEP, 3,250 kcal/kg and 16.5% for the grower diet and 3,350 kcal/kg and 15.5% for the finisher diet; HEP, 3,350 kcal/kg and 17% for the grower diet and 3,450 kcal/kg and 16% for the finisher diet.

3 Standard error of the means.

## 4 Discussion

### 4.1 Growth performance

Both dietary ME and CP levels and the ME:CP ratio could affect the growth performance of Pekin ducks in the starter phase (10, 11, 22) and grower phase (8, 12, 23). The low ME (3,150 and 3,250 kcal/kg) and CP (16.0 and 15.0%) diets significantly (*p* < 0.05) increased BWG in the present study, which indicates that too high ME and CP levels may not be necessary for Pekin ducks in the grower and finisher phase. This is not in agreement with the previous study of Liu et al. (9), who reported that increasing dietary ME (2,850, 2,950, 3,050, and 3,150 kcal/kg) and CP (16.0, 16.5, 17.0, and 17.5%) levels under the same ME:CP ratio (178) and AA profile significantly (*p* < 0.05) increased BWG linearly but significantly (*p* < 0.05) decreased F/G and FI in Pekin ducks from 15 to 40 days of age. Furthermore, the constant lysine level (1.25%) used in our study was higher than that in previous studies (0.9–1.0%), which may have led to different results (11, 12). Wen et al. (11) reported that higher dietary ME levels required greater AA requirements to compensate for lower FI in Pekin ducks, which was supported by Wu et al. (12). When surplus CP or lysine is provided, the ME level must also be increased to ensure sufficient energy for the efficient utilization of CP or lysine (8). However, we did not observe this response in the current study. The aforementioned inconsistency may be due to the higher ME level used in our study compared with previous studies. Fan et al. (23) reported a significant (*p* < 0.05) increase in BWG and a decrease in FI and F/G as dietary ME increased from 2,600 to 3,100 kcal/kg. Baéza (1) reviewed the previous studies about the ME and CP requirements of ducks in the starter and grower phases and indicated that the Pekin ducks were able to regulate the amount of energy ingested when the dietary ME level was between 2,300 and 3,100 kcal/kg and the CP level was between 16.0 and 22.0%. However, the instinct of Pekin ducks to regulate FI according to dietary energy levels was not confirmed in this study, which had higher ME levels. The growth performance response to higher ME (3,150–3,450 kcal/kg) in the grower phase differed from that observed in previous studies with relatively low ME in Pekin ducks. This may also be attributed to the fact that the 3,150 kcal/kg ME could meet the nutritional needs of Pekin ducks. The F/G in the present study was approximately 2.30–2.39. Nevertheless, a previous study (9) indicated that F/G decreased only from 2.91 to 2.60 with increasing ME (2,850–3,150 kcal/kg) and CP (16.0–17.5%) levels. In China, the dietary ME level (3,100–3,400 kcal/kg) in different phases was much higher than the recommended value for reducing F/G by commercial farms. This nutrient density strategy was demonstrated in the present study. Moreover, a two-phase feeding program in the grower phase was used for Pekin ducks (No. 4 strain) produced by the industry in China, particularly for the production of roast ducks. In the current study, the low ME (3,150 kcal/kg) and CP (16.0%) levels resulted in significantly (*p* < 0.05) higher BWG but significantly (*p* < 0.05) lower FI and F/G compared with the higher ME (3,250 and 3,350 kcal/kg) and CP (16.5 and 17.0%) levels in Pekin ducks during days 15–28. During days 29–42, the medium ME (3,350 kcal/kg) and CP (15.5%) levels led to significantly (*p* < 0.05) higher BWG and lower F/G compared with both the low and high ME and CP levels. This can provide a theoretical basis for industrial practices to improve cost-effective performance through a two-phase feeding program. To the best of our knowledge, this is the first study to investigate the effects of high ME and moderate CP levels in Pekin ducks for 2 phases. Further studies are needed to evaluate the influence of high ME and moderate CP levels in Pekin ducks during two phases.

### 4.2 Carcass traits

Pekin ducks for roasting require a high amount of subcutaneous/abdominal fat to ensure flavor after cooking (9). Subcutaneous fat is involved in determining carcass quality, particularly nutritional and sensory characteristics, in Pekin roasting ducks (3). The final BW (3.0–3.3 kg) and subcutaneous fat yield (37%) in all treatments satisfied the need for roasting Pekin ducks (2). In the present study, dietary treatments did not affect the eviscerated carcass, subcutaneous fat, left leg meat, blood, or feather yield, which was in agreement with the results of a previous study (9). The abdominal fat yield increased significantly (*p* < 0.05) with increasing ME and CP levels in our study. It is possible that the increased dietary energy may have led to the deposition of excess abdominal or carcass fat in ducks (23). Similarly, several studies have observed increased abdominal fat yield as dietary ME increased in Pekin ducks (1, 11, 23). The breast meat yield decreased significantly (*p* < 0.05) with increasing ME and CP levels in the current study. In accordance with our results, Wu et al. (12) found that the breast meat yield was significantly (*p* < 0.05) decreased, but the abdominal fat yield was significantly (*p* < 0.05) increased when the dietary ME level increased from 2,585 to 3,100 kcal/kg in Pekin ducks. On the contrary, another study by Liu et al. (9) did not observe any effect of ME and CP levels on breast meat or abdominal fat yield in Pekin ducks. The results were not always consistent. There was an interaction between dietary ME (2,820, 3,060, and 3,300 kcal/kg) and CP (15.0, 17.0, and 19.0%) levels with different ME:CP ratios on breast meat yield on day 35 (8). They reported that breast meat yield decreased in Pekin ducks as dietary ME increased, whereas breast meat yield increased as dietary CP increased. The aforementioned inconsistency may be due to the different ME:CP ratios and higher ME levels used in this study compared with previous studies. Therefore, further studies are needed to determine the effects of high ME and CP levels on the carcass traits of Pekin ducks.

### 4.3 Meat quality and taste scores

In this study, no differences were observed in meat color, shear force, or drip loss between treatments. Both meat color and carcass characteristics may strongly influence consumer choice. The flavor of Pekin ducks after roasting is very vital to consumers, especially in the era of upgrading food consumption. Low and high ME and CP levels showed significantly (*p* < 0.05) higher effects on flavor. Similarly, low and high ME and CP levels showed significantly (*p* < 0.05) a lower effect on roasting loss and a higher effect on the comprehensive score. Ao and Kim (2) reported that ducks fed with soybean oil diets had significantly (*p* < 0.05) lower roasting loss and higher comprehensive scores than those fed with duck fat and palm oil diets. This may be attributed to the fatty acid profile, which is easily modulated by the fat sources in Pekin duck diets (1, 5).

## 5 Conclusion

The present study concludes that ducks fed with the LEP diet showed the best growth performance during days 15–28 and days 15–42, while ducks fed with the MEP diet showed the best growth performance during days 29–42. Furthermore, ducks fed with LEP and HEP diets showed the lowest roasting loss and highest comprehensive score.

## Data Availability

The original contributions presented in the study are included in the article/supplementary material, further inquiries can be directed to the corresponding author.
